# Supplementary material for: Antioxidant and Antimicrobial Properties of Helichrysum italicum (Roth) G. Don Hydrosol
Source: Antibiotics (Basel). 2022 Jul 28;11(8):1017. doi: 10.3390/antibiotics11081017 (PMC9404988; doi:10.3390/antibiotics11081017)
Supplement: Supplementary file 1 [file antibiotics-11-01017-s001.zip › antibiotics-1810800-supplementary.pdf]

antibiotics

MDPI

Supplementary File

Table S1. Chemical constituents of *Helichrysum italicum* (Roth) G. Don essential oil.

No.	Compound	Area percentage (%)
1.	**α-pinene**	**5.6**
2.	α-fenchene	0.1
3.	β-pinene	0.3
4.	limonene	2.0
5.	1,8-cineol	0.5
6.	γ-terpinene	0.5
7.	linalool	0.4
8.	2-methylbutyl-2-methylbutanoate	0.3
9.	nerol	0.3
10.	**neryl acetate**	**10.8**
11.	α-copaene	2.6
12.	italicene	3.1
13.	*cis*-α-bergamotene	0.9
14.	***trans*-β-caryophyllene**	**8.4**
15.	*trans*-α-bergamotene	0.9
16.	4,6,9-trimethyldec-8-ene-3,5-dione	3.3
17.	neryl propionate	2.0
18.	γ-selinene	2.4
19.	**γ-curcumene**	**18.1**
20.	**ar-curcumene**	**5.8**
21.	**β-selinene**	**9.9**
22.	2,4,6,9-tetramethyldec-8-ene-3,5-dione	2.4
23.	**α-selinene**	**6.9**
24.	α-muurolene	0.5
25.	γ-cadinene	0.4
26.	δ-cadinene	1.7
27.	3,5,7,10-tetramethylundec-9-ene-4,6-dione	0.9

Bold means the highest percentage of the essential oil compounds.
